# Public opinion evaluation on social media platforms: a case study of High Speed 2 (HS2) rail infrastructure project

**DOI:** 10.14324/111.444/ucloe.000063

**Published:** 2023-09-08

**Authors:** Ruiqiu Yao, Andrew Gillen

**Affiliations:** 1 Civil, Environmental and Geomatic Engineering, University College London, London, UK; 2 Department of Civil and Environmental Engineering, Northeastern University, Boston, MA, USA

**Keywords:** public opinion evaluation, civil infrastructure projects, machine learning, sentiment analysis, topic modelling

## Abstract

Public opinion evaluation is becoming increasingly significant in infrastructure project assessment. The inefficiencies of conventional evaluation approaches can be improved with social media analysis. Posts about infrastructure projects on social media provide a large amount of data for assessing public opinion. This study proposed a hybrid model which combines pre-trained RoBERTa and gated recurrent units for sentiment analysis. We selected the United Kingdom railway project, High Speed 2 (HS2), as the case study. The sentiment analysis showed the proposed hybrid model has good performance in classifying social media sentiment. Furthermore, the study applies latent Dirichlet allocation topic modelling to identify key themes within the tweet corpus, providing deeper insights into the prominent topics surrounding the HS2 project. The findings from this case study serve as the basis for a comprehensive public opinion evaluation framework driven by social media data. This framework offers policymakers a valuable tool to effectively assess and analyse public sentiment.

## Introduction

Infrastructure systems lay the foundation of the economy for a nation by providing primary transportation links, dependable energy systems and water management systems to the public. In the United Kingdom (UK), the National Infrastructure Strategy 2020 reveals the determination of the UK government to deliver new infrastructure and upgrade existing infrastructure across the country to boost growth and productivity and achieve a net-zero objective by 2050 [1]. Although infrastructure projects positively affect the national economy, they can negatively impact the environment and society. For instance, they may disrupt the natural habitat of wildlife by filling up wetlands. As a result, the wildlife may have to migrate to other regions, causing problems to the ecology of certain regions [2].

Environmental impact assessments (EIA) are a critical part of the planning and delivery of large infrastructure projects. In EIA research, public participation schemes are becoming increasingly popular. O’Faircheallaigh [3] emphasised the importance of public participation in EIA decision-making processes. Social media platforms are gaining increasing ubiquity and are emerging methods for the public to participate in decision-making processes and raise environmental concerns. Thus, the research objective of this study is to evaluate the feasibility of using social media data to perform public participation analysis.

### Conventional approaches to public opinion evaluations

Public hearings and public opinion polling are the two most adopted public consultation approaches. Checkoway [4] stated some drawbacks of public hearings. For instance, the technical terms are hard to understand for the public, and participants often do not represent the actual population. As for polling, Heberlein [5] revealed that conducting polling can usually take a month or even years. As civil infrastructure projects typically have tight project timelines, there is a need for a more efficient public opinion evaluation method.

Moreover, Ding [6] argued that the data collection process is costly for conventional opinion polling. A typical 1000-participant telephone interview will cost tens of thousands of US dollars to carry out [7]. Besides conducting surveys, costs associated with data input and data analysis should also be considered [6].

Public hearings and polling are not ideal for obtaining public opinions for infrastructure projects. They can be costly, invasive and time-consuming. Therefore, researchers have drawn attention to developing an alternative method for obtaining and assessing public opinion. A new opportunity in acquiring and evaluating public opinion has emerged with the growing popularity of various social media platforms [8]. User-generated content on social media platforms provides a huge amount of data for text mining. This text data is an alternative resource for opinion evaluation toward civil infrastructure projects.

### Related work on public opinion evaluation with social media analysis

Kaplan and Haenlein [9] defined social media platforms as Internet-based applications adopting Web 2.0 (participative Web). Due to the number of active users on Facebook and Twitter, the massive amount of user-generated content provides valuable opportunities for researchers to study various social topics [10]. Moreover, with machine learning and natural language processing, researchers can perform advanced and automated algorithms on social media posts, such as sentiment analysis and topic modelling. Sentiment analysis can categorise the textual data in social media into different emotional orientations, providing an indicator of public opinion. Recent research on infrastructure project evaluation with social media analysis revealed the feasibility of using social media analysis as an alternative public opinion evaluation method.

Aldahawi [11] investigated social networking and public opinion on controversial oil companies by sentiment analysis of Twitter data. Kim and Kim [12] adopted lexicon-based sentiment analysis for public opinion sensing and trend analysis on nuclear power in Korea. Lexicon-based sentiment analysis with domain-specified dictionaries and topic modelling has also been used on public opinion data for California High-Speed Rail and the Three Gorges Project [6,8]. Lexicon-based sentiment analysis calculates the sentiment of documentation from the polarity of words [13]. In lexicon-based sentiment analysis, it is assumed that words have inherent sentiment polarity independent of their context. A user must establish dictionaries containing words with sentiment polarity to build a lexicon-based classifier. After building up the classifier, the polarity of a document is calculated in three phases: establishing word-polarity value pairs, replacing words in the document with polarity values and calculating the sentiment polarity for the document. Ding [6] tailor-made a dictionary by removing unrelated words from a positive word list. Jiang et al. [8] built a dictionary for hydro projects by integrating the Nation Taiwan Sentiment Dictionary [14], Hownet (a Chinese/English bilingual lexicon database) [15] and a hydro project-related word list. Recent research showed the practicality of implementing the lexicon-based sentiment analysis for public opinion evaluation on civil projects. The recent developments in deep learning show a promising future for public opinion evaluation.

### Recent development of natural language processing

In 2014, Bahdanau et al. [16] introduced a novel neural network architecture named attention mechanisms. Attentional mechanisms are designed to mimic cognitive perception, which computes the attention weight on input sequences so that some parts of the input data obtain more attention than the rest. In 2017, Vaswani et al. [17] published their ground-breaking research paper ‘Attention is all you need’, where they proposed an influential neural network named transformer. The transformer architecture leverages self-attention and multi-head attention to enable parallel computation. Using multiple attention heads and a self-attention mechanism, the transformer architecture can obtain different aspects of input data through learning different functions. As a result, transformer architecture can handle increased model and data size. Kaplan et al. [18] demonstrated that transformer models have remarkable scaling behaviour as model performance increases with training size and model parameters. Hence, natural language processing can benefit from large-language models, such as generative pre-trained transformer (GPT) [19,20] and Bidirectional Encoder Representations from Transformers (BERT) [21].

### Research question and main contributions

The recent developments in deep learning research motivated this study to assess how state-of-art machine learning algorithms can help public opinion evaluation on infrastructure projects. The main contributions of this study include:

1. This study proposed a hybrid transformer-recurrent neural network model for sentiment analysis, which combines the pre-trained Robustly optimised BERT approach (RoBERTa) [22] and bidirectional gated recurrent neural networks [23].
2. This study employed tweets data of High Speed 2 (HS2) as a case study, utilising it to compare the performance of the proposed RoBERTa–bidirectional gated recurrent unit (BiGRU) with baseline classifiers. Moreover, this study applied topic modelling with latent Dirichlet allocation (LDA) on tweet corpus.
3. Based on the insights from the case study results, the study proposes a public opinion evaluation framework that leverages social media data with RoBERTa–BiGRU and topic modelling. This framework provides a valuable tool for policymakers to evaluate public opinion effectively.

The rest of this article is organised as follows: the machine learning models section provides a detailed exposition of the machine learning algorithms used in this study. The case study with the High Speed 2 project section delves into the specific details and findings. This is followed by the limitations of this research and suggests potential avenues for future research. Finally, the conclusion summarises the main findings and contributions.

## Machine learning models

This section provides a comprehensive overview of implementing machine learning algorithms for public opinion evaluation. The formulation of the multinomial naïve Bayes (MNB) classifier is presented. The proposed RoBERTa–BiGRU model is then introduced, highlighting its essential components and architecture. Finally, the topic modelling technique using LDA is discussed.

### Sentiment analysis with an MNB classifier

The naïve Bayes classifier is a family of probabilistic classification models based on the Bayes theorem [24]. The term ‘naïve’ means the naïve assumption of independence among each pair of features (attributes) and class variable values [25]. More specifically, the ‘naïve’ assumption means that classifiers process the text data independently as a bag-of-words, ignoring the relationships among words, such as sequences, and only considering the word frequency in the document. The mathematical formula of the Bayes theorem Eq. (1) states that given *n* feature vectors *x*_1_,…,*x_n_* and class variable *y*, the probability distribution of *y* is:

(1) $$P(y\text{|}x_{1},\;\ldots ,x_{n})=\frac{P\;(y)\;P(x_{1},\;\ldots ,x_{n}\text{|}y)}{P(x_{1},\;\ldots ,x_{n})}$$

Because the probability distribution of feature vectors *P*(*x*_1_,…,*x_n_*) is given by the model input, the following classification rule Eq. (2) and Eq. (3) can be obtained [26]:

(2) $$P(y\text{|}x_{1},\;\ldots ,\;x_{n})\propto P(y)\overset{n}{\underset{i=1}{\prod }}P(x_{i}\text{|}y)\;$$

(3) y^=Pyargmax(y)∏i=1nP(xi|y) ,

where *P*(*y*) is the frequency distribution of *y* in the training dataset and *P*(*x_i_*|*y*) is determined by the naïve Bayes classifier assumptions. For example, the Gaussian naïve Bayes classifier assumes *P*(*x_i_*|*y*) follows a Gaussian distribution.

In the case of the MNB classifier, the multinomial distribution is parameterised by (θ_*y*1_,…, θ*_yn_*) vectors for each *y* with *n* features. θ*_yi_* indicates the probability distribution of *x_i_* under class *y* in the training set. In other words, θ*_yi_* = *P*(*x_i_*|*y*). Then, smoothed maximum likelihood estimation [27] can be used to estimate θ*_yi_*:

(4) $${\hat{\theta }}_{yi}=\;\frac{N_{yi}+\;\alpha }{N_{y}+\;\alpha n}\text{,}$$

where *N_yi_* is the number of occurrences of feature *i* for sentiment class *y*; *N_y_* is the number of occurrences of all features for *y*; and α is the smoothing prior, which is a hyperparameter to be tuned.

### Sentiment analysis with RoBERTa–BiGRU

As mentioned in the recent development of natural language processing, transformer architectures have remarkable scaling ability to handle large training data sizes and model parameters. As a result, researchers have proposed fine-tuning a pre-trained large-scale transformer model for specific downstream natural language processing tasks. This approach is referred to as transfer learning and leverages knowledge learned from the large-scale database to other downstream tasks [28]. The Bidirectional Encoder Representations from Transformers (BERT) [21] is a large language model that has state-of-the-art for natural language processing performance. The BERT model encodes text data in a bidirectionally way such that BERT can process text tokens in both left-to-right and right-to-left directions. This study used a variant of the BERT model, named the Robustly optimised BERT approach (RoBERTa) [22], because RoBERTa is pre-trained on a much larger scale of text data than BERT.

**Figure 1:**
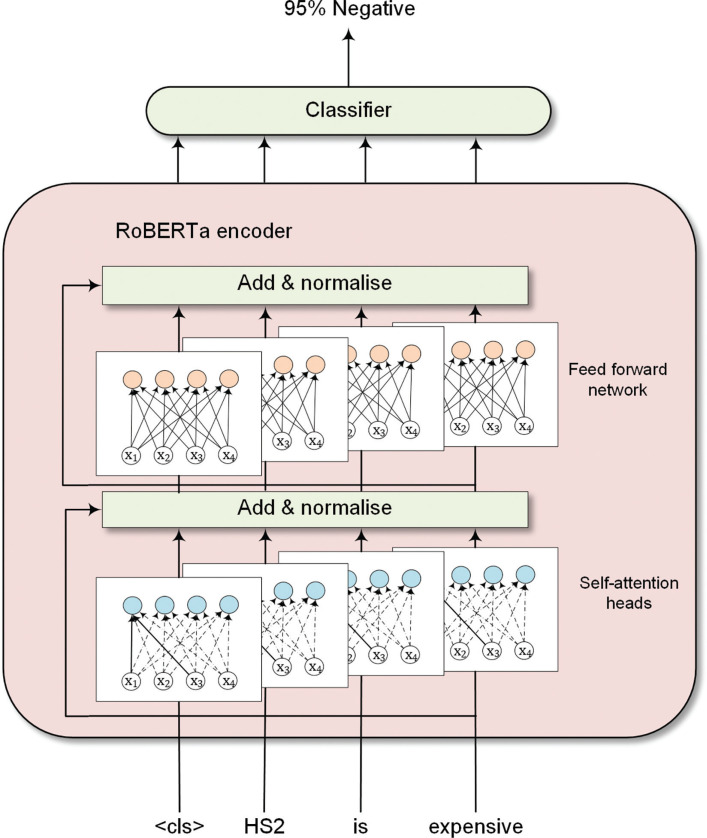
Fine-tuning RoBERTa for sentiment analysis.

Details of fine-tuning the RoBERTa model for sentiment analysis are shown in Fig. 1. RoBERTa used similar transformer architecture as BERT. The input token sequence is passed to multiple self-attention heads, followed by a layer normalisation [29]. The normalised data is subsequently sent to feed-forward networks and a second layer normalisation. Figure 1 shows the transformer architecture of a single encoder layer. The RoBERTa model contains multiple encoders based on model preference. A RoBERTa encoder’s hidden states can then be fed into a classifier for classification tasks. Noticeably, the ‘<cls>’ token indicates the global representation of input text [28].

The classifier can be different neural network architectures, such as feedforward neural networks (FNN) or recurrent neural networks (RNN). The long short-term memory (LSTM) architecture is a prevalent choice as the classifier [30]. The LSTM introduced internal states and gates in addition to RNN to process information in sequenced data [31]. The GRU architecture, proposed by Cho [23] in 2014, is a streamlined adaptation of LSTM architecture which retains internal states and gating mechanisms. This study adopted the GRU architecture as a classifier from RoBERTa outputs because gated recurrent unit (GRU) has a faster computation speed than LSTM with comparable performance [32].

The GRU model consists of two internal gates: a reset gate and an update gate. The reset gate determines the extent to which information from the previous state is retained, while the update gate controls the proportion of the new state that replicates the old state. The mathematical formulate of the reset gate and update gate are:

(5) $$R_{t}=\sigma (W_{ir}X_{t}+b_{ir}+W_{hr}H_{t-1}+b_{hr})\;$$

(6) $$Z_{t}=\;\sigma (W_{iz}X_{t}+b_{iz}+W_{hz}H_{t-1}+b_{hz})\;$$

(7) $$\sigma (x)=\frac{1}{1+\exp(-x)},$$

where $X_{t}\in {\mathbb{R}}^{n\times d}$ is a minibatch input of a memory cell (*n* is the number of sample and *d* is the dimension of features); $H_{t-1}\in {\mathbb{R}}^{n\times h}$ is the hidden state of the previous step (*h* is the number of hidden units of a GRU memory cell); ***W****_ir_*, $W_{hr}\in {\mathbb{R}}^{d\times h}$ and ***W****_iz_*, $W_{hz}\;\in {\mathbb{R}}^{h\times h}$ are model weights; and *b_ir_*, *b_hr_*, *b_iz_*, and *b_hz_* are model bias parameters. The reset gate $R_{t}\in {\mathbb{R}}^{n\times h}$ and update gate $Z_{t}\in {\mathbb{R}}^{n\times h}$ are computed based on Eq. (5) and Eq. (6). In other words, two gates are fully connected layers with sigmoid activation function Eq. (7).

The reset gate is designed to yield a candidate hidden state $N_{t}\in {\mathbb{R}}^{n\times h}$ with Eq. (8) and tanh activation function Eq. (9). The influences of previous information ***H***_*t*−1_ in Eq. (8) is reduced by the Hadamard product of ***R****_t_* and ***H***_*t*−1_. The candidate hidden state ***N***_*t*_ is then passed to Eq. (10) to calculate the new hidden state ***H****_t_*, in which the update gate ***Z****_t_* controls the degree to which ***H****_t_* resembles ***N***_*t*_.

(8) $$N_{t}=\tanh(W_{in}X_{t}+b_{in}+R_{t}⊙(W_{hn}H_{t-1}+b_{hn}))$$

(9) $$\tanh(x)=\frac{1-\exp(-2x)}{1+\exp(2x)}\;$$

(10) $$H_{t}=(1-Z_{t})⊙N_{t}+Z_{t}⊙H_{t-1},$$

where $W_{in}\;\in {\mathbb{R}}^{d\times h}$ and $W_{hn}\in {\mathbb{R}}^{h\times h}$ are model weights; *b_in_* and *b_hn_* are bias parameters; and ⊙ is the Hadamard product, which is also referred to as the element-wise product.

**Figure 2:**
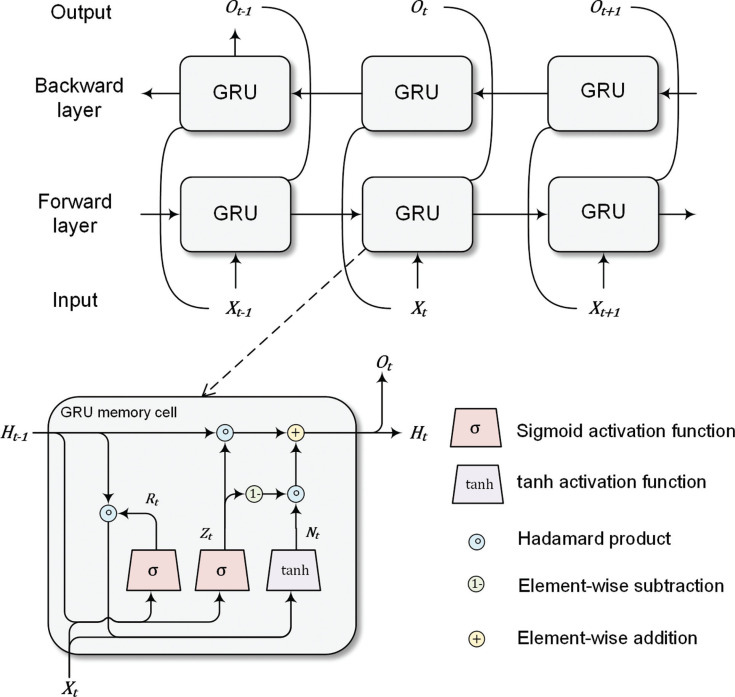
Bidirectional GRU model.

Similar to the bidirectional setting of BERT, a two-layer GRU is also able to process the text data bidirectionally with a forward layer and a backward layer, as shown in Fig. 2. The hidden state of the forward layer and backward layer is denoted as $\vec{H_{t}}\in {\mathbb{R}}^{n\times h}$ and $\overset{\leftarrow }{H_{t}}\in {\mathbb{R}}^{n\times h}\text{.}$ The forward layer hidden states $\vec{H_{t}}$ is then multiplied with a dropout rate δ, which is a Bernoulli random variable with δ probability of being 0. The output of a GRU is a concatenate of $\vec{H_{t\_\delta }}$ and $\overset{\leftarrow }{H_{t}}$ with dimension *n* × 2*h*.

The RoBERTa model can be fine-tuned by optimising the loss function of the above-mentioned bidirectional GRU and connecting the output of a bidirectional GRU with a fully connected layer.

The loss function to be optimised in GRU is a cross entropy function [33]. Moreover, the fully connected layer uses the softmax activation function Eq. (11):

(11) $$s(x_{i})=\frac{e^{x_{i}}}{\sum_{j=1}^{n}e^{x_{j}}},$$

where *n* is the number of sentiment classes. The fully connected layer converts the hidden states of the bidirectional GRU to the probability of each sentiment class.

**Figure 3:**
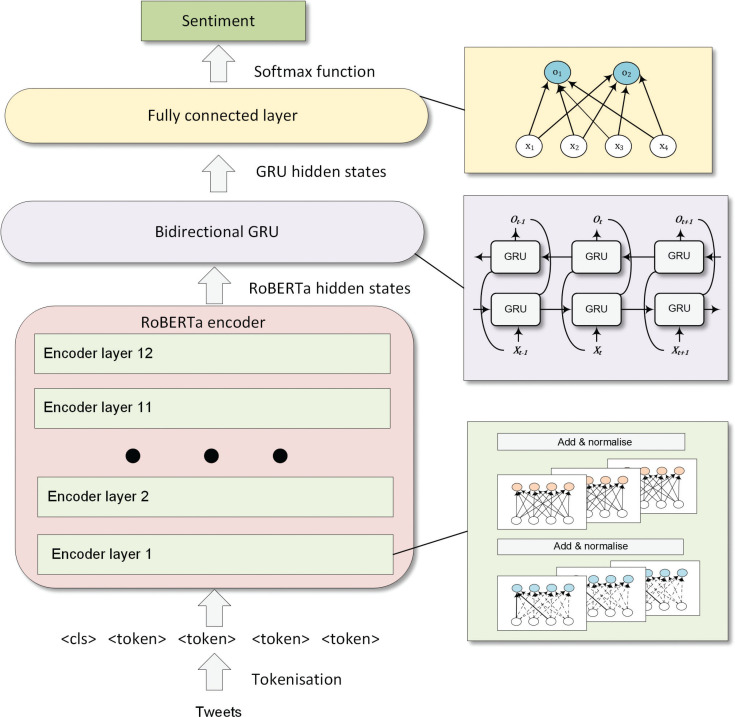
Structure of RoBERTa–BiGRU for sentiment analysis.

Figure 3 demonstrates the complete structure of the RoBERTa–BiGRU model. Firstly, tweets are tokenised with the RoBERTa tokeniser. Then, the tokens are passed to 12 encoders with multiple self-attention heads to obtain 768 tweets’ hidden representations. The tweets’ hidden representations can then be allocated to sentiment classes through a bidirectional GRU and fully connected layer.

### Topic modelling with LDA

Deerwester et al. [34] proposed a latent semantic indexing method for topic modelling, applying singular value decomposition (SVD) to derive the latent semantic structure model from the matrix of terms from documents. SVD is a linear algebra technique to decompose an arbitrary matrix to its singular values and singular vectors [35]. Blei et al. [36] introduced LDA, which is a general probabilistic model of a discrete dataset (text corpus).

LDA is a Bayesian model, which models a document as a finite combination of topics. Each topic is modelled as a combination of topic probabilities. For example, an article that talks about the structural design of a building complex may have various topics, including ‘structural layout’ and ‘material’. The topic ‘structural layout’ may have high-frequency words related to structural design, such as ‘beam’, ‘column’, ‘slab’ and ‘resistance’. Also, the ‘material’ topic may have the words ‘concrete’, ‘steel’, ‘grade’ and ‘yield’. In short, a document has different topics with a probabilistic distribution, and each topic has different words with a probabilistic distribution. Human supervision is not required in LDA topic modelling, as LDA only needs a number of topics to perform an analysis.

Topic modelling with LDA has a wide range of applications in research. Xiao et al. [37] used LDA variant topic modelling to uncover the probabilistic relationship between adverse drug reaction topics. Xiao et al. found that the LDA variant topic modelling has higher accuracy than alternative methods. Jiang et al. [8] showed the feasibility of LDA topic modelling to extract topics about the Three Gorges Project on a Chinese social media platform. Apart from focusing on extracting terms from the textual corpus, topic modelling is another trend-finding tool, as it will reveal the relationship between topics. Chuang et al. [38] proposed a method to visualise topics with circles in a two-dimensional plane, whose centre is determined by the calculated distance between topics. The distance is calculated using Jenson–Shannon divergence, and principal components analysis determines the size of the circle [39].

## Case study with the HS2 project

This section provides implementation details of sentiment classifiers and topic modelling methods for the HS2 case study. First, the background of the HS2 project is presented, offering insights into the rail infrastructure project. This is followed by an explanation on data collection and processing, detailing the methods employed to gather social media data related to HS2. Then the evaluation metrics used to assess the performance of sentiment classifiers are presented, enabling a thorough examination of sentiment classification models. The following two sections show the results of sentiment analysis and topic modelling, respectively. Finally, a framework for evaluating public opinion based on social media data is introduced.

### Background on the HS2 project

**Figure 4:**
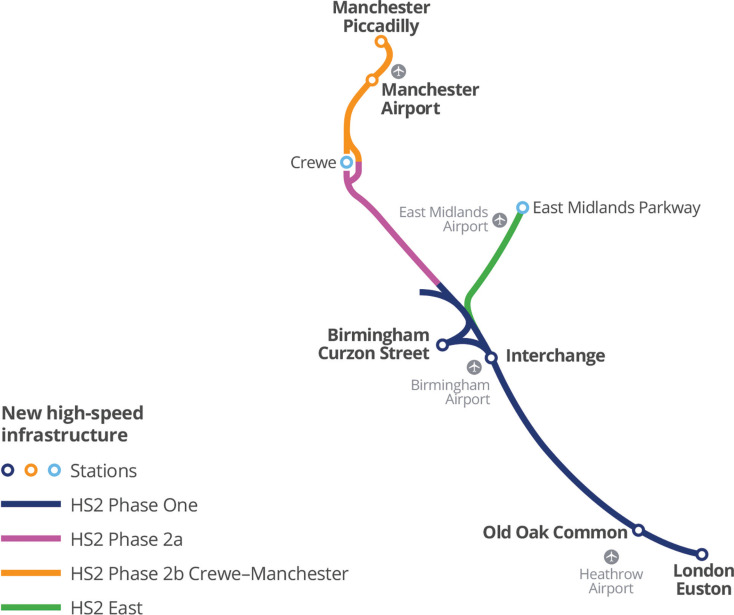
HS2 infrastructure map [41].

The transportation demand for the UK railway network has steadily grown over the past decades. According to the Department for Transport [40], rail demand has doubled since 1994–1995, with a rising rate of 3% every year. Therefore, the HS2 programme is proposed to construct a new high-speed and high-capacity railway, aiming to boost the economy in the UK, improve connectivity by shortening journey time, provide sufficient capacity to meet future railway network demand and reduce carbon emission by reducing long-distance driving. Figure 4 shows that HS2 will connect London, Leeds, Birmingham and Manchester, joining the existing railway infrastructure to allow passengers to travel to Glasgow, Newcastle and Liverpool [41].

### Data preparation

The collection of HS2-related tweets was carried out using Twitter application programming interfaces (API). Specifically, tweets that containing the hashtags ‘#HS2’ and ‘#HighSpeed2’ were collected. However, the number of collectable tweets is constrained by the limitations imposed by the Twitter API, which restricts the collection to under 10,000 tweets. Thus, the total number of tweets collected was 8623 tweets. The tweets were sampled over a 5-year period from 2017 to 2020. The tweet distribution across the years is: 2017 (1544 tweets), 2018 (1130 tweets), 2019 (2909 tweets) and 2020 (3040 tweets). Noticeably, the tweets collected were in an extended mode, allowing the retrieval of the complete text, surpassing the 140-character limit.

Data preprocessing involves cleaning and preparing data to increase the accuracy and performance of text-mining tasks, such as sentiment analysis and topic modelling. Tweet text data tend to contain uninformative text, such as URL links, Twitter usernames and email addresses. For MNB and lexicon-based classifiers, the stop words need to be removed. To be more specific, stop words are words that do not have sentiment orientation, such as ‘me’, ‘you’, ‘is’, ‘our’, ‘him’ and ‘her’. As each word in text data is treated as a dimension, keeping stop words and uninformative text will complicate the text mining by making text mining a high dimension problem [42]. Other text preprocessing techniques for MNB and lexicon-based classifiers include text lowercasing and text stemming. Noticeably, the transformer architectures do not require removing stop words, lowercasing and text stemming, as transformers are able to handle the implied information in stop words.

Upon conducting a manual inspection of collected tweets, the number of tweets expressing positive sentiment was significantly lower than those with negative or neutral sentiment. The sentiment classification task is set to binary to address the imbalance issue. The task was designed to classify tweets as either having negative sentiment or non-negative sentiment (including neutral and positive sentiments). A set of 1400 tweets was carefully annotated to train classifiers in this case study. Within this annotated dataset, 700 tweets were labelled as negative sentiment, while the remaining 700 tweets were labelled as non-negative sentiment. To access the annotated training tweets, a GitHub link is provided in the Open data and materials availability statement, facilitating transparency and reproducibility of this study. The annotated tweets were split into 70% training dataset (980 tweets) and 30% validation dataset (420 tweets).

### Sentiment analysis results

Three sentiment classifiers were used in this case study: (1) VADER [43], a rule-based lexicon sentiment classifier. (2) An MNB classifier, which is built following details in the background on the HS2 project. (3) A RoBERTa–BiGRU model that is developed based on the architecture presented in the data preparation section. The model details of each classifier are shown in Table 1. The hyperparameters in MNB and RoBERTa–BiGRU, such as smoothing priors *α*, batch size, hidden units and dropout rate, were tuned by a grid search. The RoBERTa–BiGRU model was trained on a Tesla T4 GPU on Google Colab with a total training time of 2421.23 s for 100 epochs.

**Table 1.** Model details of each classifier

Name	Model parameters
VADER	Rules specified in [43]
MNB	Smoothing priors: *α* = 0.1
RoBERTa–BiGRU	Batch size: 16 Hidden units: 256 Dropout rate: 0.5 Optimiser: AdamW Learning rate: 2 × *e*^–6^ Epoch: 100

The performances of three classifiers were evaluated with accuracy and receiver operating characteristic (ROC) curve. Accuracy, as shown in Eq. (12), measures the accuracy of the classifier with all correctly identified cases overall. A ROC curve plots the true positive rate, as shown Eq. (13), along the *y* axis and the false positive rate, as shown in Eq. (14), along the *x* axis. An ROC curve shows the graphical interpretation of gain (true positive rate) and loss (false positive rate) [44]. The area under the curve (AUC) score calculates the total area under the ROC curve. The AUC score quantitatively evaluates the performance of a classifier, which represents the possibility that a random positive datapoint ranks higher than a random negative datapoint [45].

(12) $$\text{accuracy }=\;\frac{TP+TN}{TP+TN+FP+FN}$$

(13) $$\text{true positive rate (recall) }=\;\frac{TP}{TP+FN}$$

(14) $$\text{false positive rate }=\;\frac{FP}{FP+TN},$$

where *TP* = true positive, *TN* = true negative, *FP* = false positive and *FN* = false negative.

**Figure 5:**
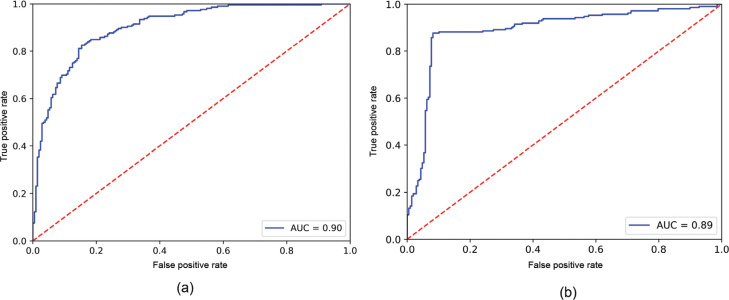
(a) ROC curve for MNB classifier. (b) ROC curve for RoBERTa–BiGRU.

Table 2 demonstrates the accuracy of each sentiment classifier. The lexicon-based VADER has the lowest accuracy (70.24%) among the three classifiers. MNB and RoBERTa–BiGRU show better accuracy performance than VADER, whereas MNB and RoBERTa–BiGRU have increased accuracies of 12.38% and 19.28%, respectively. MNB and RoBERTa–BiGRU are then compared with respect to the AUC scores. MNB has an AUC score of 0.9023, while RoBERTa–BiGRU has a slightly lower AUC score of 0.8904. Both MNB and RoBERTa–BiGRU have an AUC score of around 0.9, which indicates that both models have a high level of ability to classify tweet sentiment. Noticeably, Fig. 5(b) has a much steeper curve. The steeper curve means that RoBERTa–BiGRU can achieve higher recall with a low FP rate, which is desirable behaviour in sentiment analysis. As a result, RoBERTa–BiGRU has the best performance in terms of both accuracy and the ROC curve. Thus, RoBERTa–BiGRU was used for sentiment analysis with all collected tweets.

**Table 2.** Model accuracy performance

Name	Accuracy
VADER	70.24%
MNB	82.62%
RoBERTa–BiGRU	89.52%

**Figure 6:**
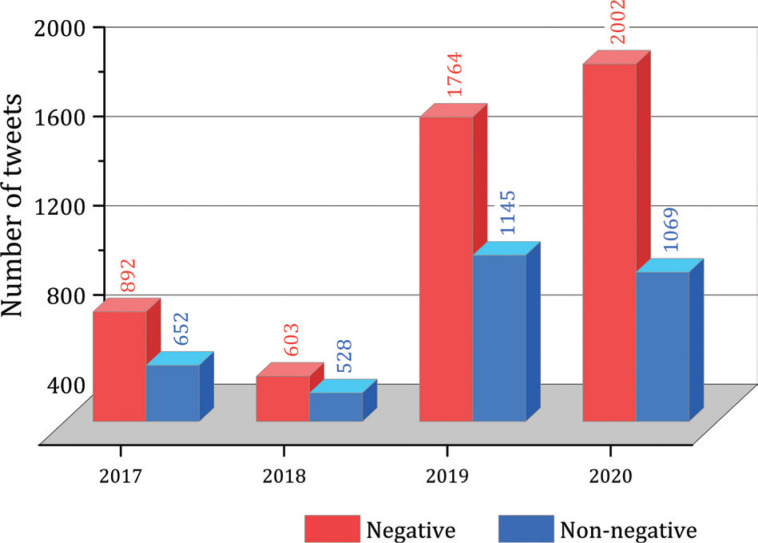
Sentiment analysis results for 2017–2020.

Figure 6 shows the sentiment distribution of HS2-related tweets from 2017 to 2020. Notably, there was a substantial increase in the number of tweets in 2019, indicating a heightened presence of the HS2 project in social media discussions during and after that year. Moreover, it is worth mentioning that the majority of tweets collected across all time periods exhibited a negative sentiment. Specifically, negative tweets accounted for 57.77% in 2017, 53.32% in 2018, 60.64% in 2019 and 65.19% in 2020.

The substantial proportion of negative tweets in all periods indicates a prevailing negative sentiment among the public regarding HS2, highlighting the importance for policymakers and decision-makers to take this sentiment into consideration. However, it is essential to approach these findings with caution. While the high percentage of negative tweets may raise concerns, it is crucial to note that this alone does not necessarily imply a public relationship emergency for HS2. It is worth acknowledging that certain Twitter users might repeatedly express their negative sentiment towards HS2 [46], potentially influencing the overall sentiment distribution. Given the sentiment analysis results, it is important to uncover the key topics within the tweets discussions, necessitating the application of topic modelling.

### Topic modelling results

The tweets dataset is then classified using the RoBERTa–BiGRU model into two collections: negative corpus and non-negative corpus. Each collection was performed individually with topic modelling and visualisation. Topic modelling with LDA was performed with genism, a collection of Python scripts developed by Rehurek and Sojka [47]. We used pyLDAvis, a Python library, for visualising topics such that we could determine the most suitable number of topics. Several models were constructed with a number of topics ranging from 3 to 20. We selected five as the number of topics through manual inspection of term distribution and topic relevance.

#### Negative tweets corpus

Table 3 shows major topics in the negative corpus. Topic 1 is the largest topic and accounts for 35.3% of the negative corpus. Topic 1 contains words such as ‘need’, ‘money’, ‘nhs’, ‘badly’ and ‘billion’. These words express the negative sentiment on HS2 budget spending. These tweets criticise the over-spending of HS2 and argue that the money should be invested in the National Health Service (NHS) rather than HS2. Topic 2 and Topic 4 have a similar focus. Topic 2 has words such as ‘government’, ‘protester’ and ‘social’, and Topic 4 include words such as ‘stophs2’, ‘petition’, ‘media’ and ‘political’. Both Topic 2 and Topic 4 discuss the campaign to stop HS2 project by a petition. Topic 3 and Topic 5 show some relevance. Topic 3 contains ‘stop’, ‘please’, ‘trees’, ‘contractors’, ‘changed’ and ‘essential’, which raises environmental concerns about construction work on woodlands. Topic 5 also discusses the environmental issues with the words ‘construction’, ‘damage’ and ‘destroy’.

**Table 3.** Topics in negative corpus

Topic number	Terms	Topic percentage
1	Borisjohnson, hs2, work, time, need, money, say, nhs, use, uk, course, amp, transport, nt, cancel, even local, badly, billion, ancient, public, needed, boris, way, think, country, rishisunak, trains, know	35.3%
2	Rail, government, going, still, protesters, like, news, case, go, social, could, economic, train, people, home, London, times, business, ltd, working, travel, back, road, north, sense, says, dont	24.2%
3	Stop, post, mps, please, another, anti, away, seems, trees, make, already, without, contractors, may, changed, control, steeple, long, big, bill, sign, essential, protest, claydon, likely, means, yet, billions, station, caught	13.9%
4	Sopths2, workers, petition, sites, via, take, destruction, ever, change, media, track, year, ukparliament, least, investment, everyone, account, despite, find, continue, political, wants, white, along, british, longer, evidence, called, massive, elephant	13.6%
5	Report, scrap, construction, costs, last, end, law, latest, true, tax, first, damage, full, job, trident, nesting, figures, wonder, share, read, unnecessary, questions, destroy, failed, coming, vital	13.1%

#### Non-negative tweets corpus

Table 4 shows topics in a non-negative corpus. Topic 1 includes words such as ‘new’, ‘railway’, ‘good’, ‘midlands’ and ‘important’, where tweets express positive sentiment on HS2 by mentioning the positive effect on the Midlands. A similar result can be found in Topic 3, which includes words such as ‘planning’, ‘Manchester’, ‘airport’, ‘benefit’, ‘better’. Topic 3 highlights that the transportation infrastructure in Manchester could benefit from the HS2 project. Topic 2 discusses the business case of HS2 with words such as ‘project’, ‘business’, ‘build’, ‘network’ and ‘industry’. Topics 4 and 5 both discuss potential improvements on the accessibility to the airport with words ‘heathrow’, ‘airports’, ‘opportunities’. Overall, the LDA topic modelling showed good execution on obtaining key topics from the tweet corpus.

**Table 4.** Topics in non-negative corpus

Topic number	Terms	Topic percentage
1	Work, new, project, one, railway, station, first, time, may, ever, people, plans, common, good, midlands, find, watch, still, well, way, may, could, largest, part, back, important, day	35.4%
2	Construction, hs2ltd, rail, post, projects, train, business, build, track, road, read, network, phase, industry, latest, leaders, think, green, big, please, works, air, know, local, year, along	24.4%
3	High, speed, need, old, north, planning. would, capacity, built, engineering, course, Manchester, building, another, plan, recent, airport, must, benefit, needs, evidence, better, needed, chief, funding	15.9%
4	Government, news, trains, us, would, home, two, heathrow, cost, start, railways, service, suppliers, roads, update, every, keep, seems, question, longer, join, money	13.3%
5	Stations, use, lake, community, following, scheme, economic, really, opportunities, spending, committee, supply, benefits, due, chain, role, early, daily, fund, freight, article, essential, airports	11.1%

### Proposed public opinion evaluation framework using social media data

The case study results showed that the RoBERTa–BiGRU and LDA topic modelling have a good performance in evaluating public opinion on HS2 with tweet data. Hence, a public opinion evaluation framework using social media data is proposed to facilitate the decision-making of policymakers.

**Figure 7:**
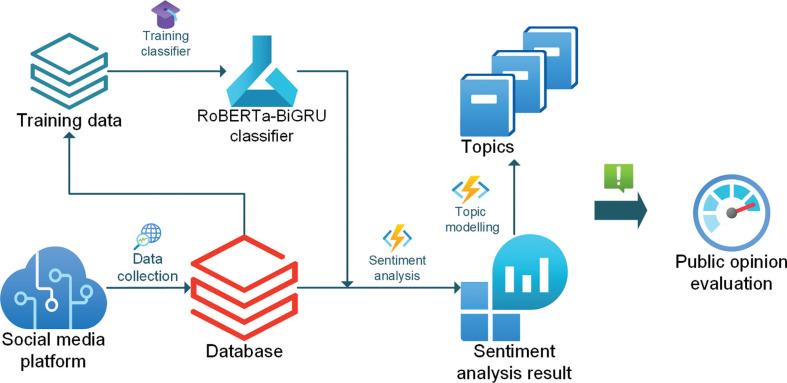
Public opinion evaluation framework.

Figure 7 presents the comprehensive public opinion evaluation framework that utilises social media data. The process begins by collecting social media data, such as tweets, and storing them in a database. Subsequently, the social media data is processed through sentiment annotation, which involves labelling the data to create training sets. These training sets are then utilised for training a sentiment classifier called RoBERTa–BiGRU. Once the RoBERTa–BiGRU sentiment classifier is trained, it is employed to categorise social media tweets into predefined sentiment labels. Additionally, leveraging LDA topic modelling, the framework extracts key topics from the social media data. Policymakers can subsequently utilise the sentiment analysis results and key topics to evaluate public opinion regarding infrastructure projects.

## Limitation and future research direction

### Human factors in annotating tweets sentiments

Researchers usually assign multiple annotators (3 to 5) to tag the sentiment orientation to minimise the influence of human annotators [48]. However, in our study, all the training data was tagged by one annotator. As a result, the human factor may have affected the accuracy of the sentiment classifier. The future application of fine-tuning sentiment classifiers could benefit from multiple annotators.

Another impact of human factors could be different sentiment interpretations. For example, the following tweet may be tagged with different sentiment orientations. ‘#HS2 is a £100bn scheme to have slightly shorter journey times from Manchester and Birmingham to London, thereby solving Britain’s biggest ever problem.’ One annotator can argue that there are positive sentiment signs (shorter journey time and solving problems). In contrast, another annotator could also argue that the tweet used a sarcastic tone to express a negative sentiment towards over budget issue of HS2.

### Topic modelling challenges

Text documents are combinations of probabilistic distributions of topics, and each topic is a probabilistic distribution of words. However, tweets are short microblogs with character limitations (280 characters), which usually contain one topic. Therefore, LDA may have problems in calculating the probabilistic distribution of topics in tweets data. The performance of tweets topic modelling could be improved with the neural optic models, leveraging deep generative models [49]. Future research on public opinion evaluation with social media data could use Bayesian networks. In particular, gamma-belief networks showed promising results in yielding structure topics [50].

## Conclusion

This study utilised tweets data from the HS2 project as a case study. The tweets data were used to compare the performance of the proposed RoBERTa–BiGRU model with MNB and VADER. RoBERTa–BiGRU showed the best performance in terms of accuracy and ROC curves. Additionally, the study employs LDA to uncover key topics within the tweet corpus. This analysis enhances understanding of the prominent themes surrounding the HS2 project. The insights derived from the HS2 case study results lay the foundation for a public opinion evaluation framework. This framework, driven by social media data, is an invaluable tool for policymakers to evaluate public sentiment effectively. Overall, this study contributes to the field of public opinion evaluation by introducing a hybrid model, presenting a comprehensive case study analysis, and proposing a practical framework for public opinion evaluation.

## Data Availability

The datasets generated during and/or analysed during the current study are available in the repository: https://github.com/RY7415/OpinionAnalysisSocialMedia. This includes the collected data (anonymised) and the Python source code.
